# P-767. Unveiling Tuberculosis: A Perspective on Active vs. Subclinical Pulmonary Tuberculosis

**DOI:** 10.1093/ofid/ofae631.962

**Published:** 2025-01-29

**Authors:** Diana D D Cardenas-Maldonado, Thomas Chevalier, Erica Herc, Odaliz Abreu Lanfranco, Geehan Suleyman

**Affiliations:** Henry Ford Hospital, Farmington Hills, Michigan; Henry Ford Health System, Detroit, Michigan; Henry Ford Hospital, Farmington Hills, Michigan; Henry Ford Health System, Wayne State University School of Medicine, Detroit, MI; Henry Ford Health, Detroit, Michigan

## Abstract

**Background:**

Despite being preventable and curable, tuberculosis (TB) remains one of the world’s leading infectious disease killers with 1.5 million people dying each year worldwide. The U.S. has one of the world’s lowest rates of TB; however, cases increased 16% from 2022 to 2023. Although risk factors and outcomes associated with TB have been described, reports characterizing TB disease in the U.S. are limited.

**Figure:**
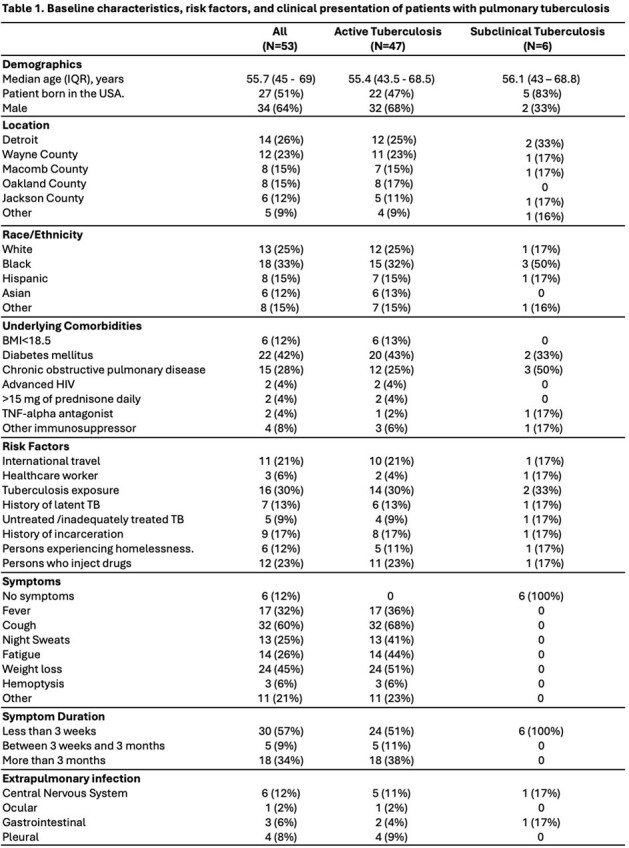

IQR: interquartile; USA: United States of America; BMI: Body Mass Index; HIV: human immunodeficiency virus; TNF-alpha inhibitors: Tumor necrosis factor-alpha inhibitors; TB: tuberculosis.

**Methods:**

Retrospective case series of patients with TB disease at Henry Ford Health in Southeast Michigan from Jan 2019-Dec 2023. Demographics, risk factors, clinical manifestations, treatment, and outcomes were evaluated.  Active and subclinical TB were compared. Active TB was defined as positive imaging and/or microbiologic assay with clinical TB-related symptoms, and subclinical TB as positive imaging and/or microbiologic assay without symptoms.

**Figure:**
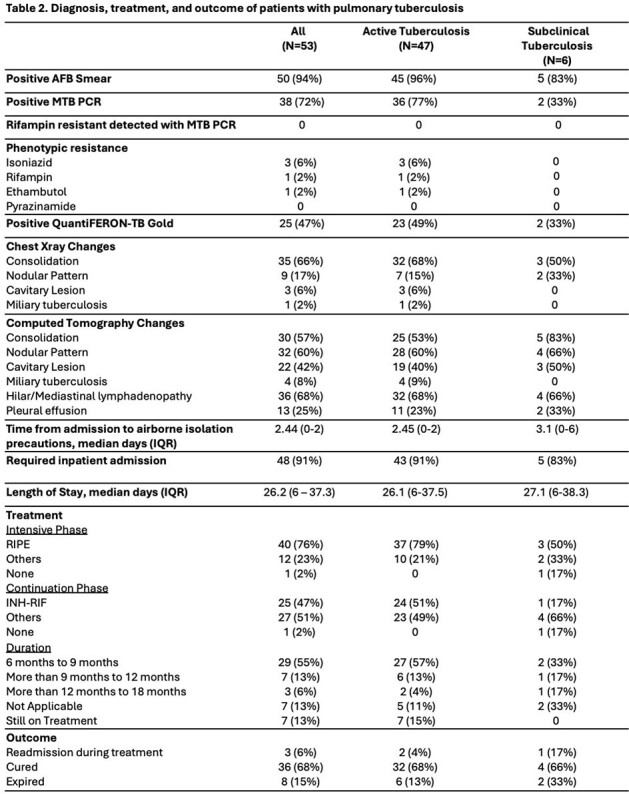

AFB: Acid-Fast-Bacilli; MTB: Mycobacterium tuberculosis; PCR: polymerase chain reaction; IQR: interquartile; TB: tuberculosis; RIPE: Rifampin, Isoniazid, Pyrazinamide, Ethambutol; INH: Isoniazid; RIF: Rifampin.

**Results:**

53 patients were included, of whom 18 (33%) were black and 34 (64%) were male with a median age of 55 years (Table 1). Almost half were born in the U.S., and a quarter lived in Detroit. Prior TB exposure (30%), international travel (21%), persons who inject drugs (23%), and incarceration (17%) were common risk factors. 47 (89%) patients had active TB, and cough (68%) was prevalent. Consolidation and nodular patterns were frequently seen on imaging; cavitation occurred in 42% of cases. All had sputum smear microscopy and culture ordered; MTB PCR was obtained in 72% of cases (Table 2). Both were more likely to be positive in those with active TB. Most patients required inpatient admission; median time to airborne isolation precautions was longer in the subclinical group. Length of stay was prolonged in both groups. All patients with active TB received treatment, whereas 83% were treated in the subclinical group. Most were treated for at least 6 months. Among those completing treatment, 68% achieved cure and 15% expired.

**Conclusion:**

In this large cohort of TB patients, almost half were US-born and a third had a known TB exposure. Although less common, subclinical TB contributed to 11% of cases. Despite treatment, clinical cure was suboptimal with a 15% mortality. Early recognition and treatment of latent and active TB are critical disease control and prevention strategies.

**Disclosures:**

**All Authors**: No reported disclosures

